# TP53-binding protein variants and breast cancer risk: a case-control study

**DOI:** 10.1186/bcr1038

**Published:** 2005-05-06

**Authors:** Bernd Frank, Kari Hemminki, Justo Lorenzo Bermejo, Rüdiger Klaes, Peter Bugert, Barbara Wappenschmidt, Rita K Schmutzler, Barbara Burwinkel

**Affiliations:** 1 Division of Molecular Genetic Epidemiology, German Cancer Research Center (DKFZ), Heidelberg, Germany; 2 Department of Biosciences at Novum, Karolinska Institute, Huddinge, Sweden; 3 Institute of Human Genetics, University of Heidelberg, Heidelberg, Germany; 4 Institute of Transfusion Medicine and Immunology, Red Cross Blood Service of Baden-Württemberg-Hessia, University of Heidelberg, Faculty of Clinical Medicine, Mannheim, Germany; 5 Division of Molecular Gynaeco-Oncology, Department of Gynaecology and Obstetrics, Clinical Center University of Cologne, Germany

## Abstract

**Introduction:**

The TP53-binding protein (53BP1) has been shown to influence TP53-mediated transcriptional activation, thus playing a pivotal role in DNA damage signalling. Genetic aberrations in *TP53 *and in *ATM *and *CHEK2 *predispose to cancer. We have therefore examined the effects of 53BP1 single nucleotide polymorphisms (D353E, G412S, and K1136Q) and the novel *53BP1 *6bp deletion (1347_1352delTATCCC) on breast cancer risk.

**Methods:**

Allelic discrimination was performed to investigate the frequencies of 53BP1 D353E, G412S, and K1136Q and of 1347_1352delTATCCC in 353 patients with breast cancer and 960 control individuals.

**Results:**

No significant association of 53BP1 D353E, G412S, or K1136Q with breast cancer risk was detected. *53BP1 *1347_1352delTATCCC, leading to the loss of an isoleucine and a proline residue, showed a nonsignificant inverse association with breast cancer risk (odds ratio = 0.61, 95% confidence interval = 0.22 to 1.68, *P *= 0.34).

**Conclusion:**

The lack of association casts doubt on the putative effects of D353E, G412S, and K1136Q on breast cancer risk. Investigating a larger study cohort might elucidate the influence of the 6bp deletion 1347_1352delTATCCC. Studying the functional effect and the impact of this variant on the risk of other cancers may be revealing.

## Introduction

The TP53-binding protein (53BP1), a conserved nuclear protein, was initially identified to interact with the DNA-binding domain of TP53, thus enhancing TP53-mediated transcriptional activation [1,2]. In response to exogenous exposure to ionising radiation, 53BP1 becomes hyperphosphorylated and rapidly localises to sites of DNA double-strand breaks, demonstrating its determining role in DNA damage signalling [3,4]. 53BP1-deficient mice exhibit growth retardation, high radiation sensitivity, and tumour development – features that are indicative of a defective DNA damage response [5]. 53BP1 is involved in the phosphorylation of various ataxia telangiectasia mutated protein (ATM) substrates such as cell cycle checkpoint kinase 2 (CHEK2) [3,6]. Mutations in ATM, CKEK2, and its substrate, TP53, have been shown to predispose to cancer [6-9]. Therefore, we selected *53BP1 *as an attractive candidate gene for breast cancer susceptibility.

This is the first study to investigate the effects of the 53BP1 single nucleotide polymorphisms (SNPs) D353E (1059C>G), G412S (1234G>A), and K1136Q (3406A>C) on breast cancer risk, analysing 353 German patients with breast cancer and 960 controls. 53BP1 D353E, G412S, and K1136Q showed no association with breast cancer risk. In addition, we detected a novel, very rare *53BP1 *6bp deletion (1347_1352delTATCCC) showing an inverse association with breast cancer risk (age-adjusted odds ratio (OR) = 0.61, 95% confidence interval (CI) = 0.22 to 1.68), lacking significance (*P *= 0.34).

## Materials and methods

### SNP verification

A randomly chosen set of 23 German patients with familial breast cancer was initially screened for annotated *53BP1 *SNPs (dbSNP database; NCBI (National Center for Biotechnology Information)) by DNA sequencing. Sequencing primers are available upon request. The initial analysis included *53BP1 *exons 9, 11, and 17, harbouring three reported nonsynonymous polymorphisms (D353E: rs560191; G412S: rs689647; and K1136Q: rs2602141). When sequencing exon 11, we additionally detected the 6bp deletion 1347_1352delTATCCC. All validated variants were chosen for further analyses using a large cohort of breast cancer patients.

### Subjects

The breast cancer patients were 353 unrelated German women (mean age 44.8 years, range 21 to 80 years) who were negative for *BRCA1 *and *BRCA2 *mutations. In accordance with the German Consortium for Hereditary Breast and Ovarian Cancer, they were classified into six categories based on family history: (A1) families with two or more breast cancer cases including two cases with onset below the age of 50 (39.3% of analysed cases); (A2) families with at least one male breast cancer case (0.9%); (B) families with at least one breast cancer and one ovarian cancer case (16.2%); (C) families with at least two breast cancer cases including one case diagnosed before the age of 50 (33.5%); (D) families with at least two breast cancer cases comprising two cases diagnosed after the age of 50 (5.5%); (E) single cases of breast cancer diagnosed before the age of 35 (4.6%) [10]. They were collected during the years 1997 to 2004 through the Institute of Human Genetics (Heidelberg, Germany) and the Department of Gynaecology and Obstetrics (Cologne, Germany). The control series included 960 blood donors (mean age 30.5 years, range 18 to 67 years) collected by the Institute of Transfusion Medicine and Immunology (Mannheim, Germany) having the same ethnic background as the breast cancer patients. Both study populations have been described earlier [11]. The study was approved by the Ethics Committee of the University of Heidelberg (Heidelberg, Germany).

PCR amplification and sequencing were performed as previously described [11]. Conditions are available on request.

### Genotyping

53BP1 polymorphisms D353E, G412S, and K1136Q were analysed using TaqMan allelic discrimination. TaqMan assays were performed in a reaction volume of 10 μl comprising 5ng of genomic DNA, each probe at 50 nM, each primer at 225 nM, and 1× Universal Master Mix with the following amplification conditions: 2 min at 50°C, 10 min at 95°C and 35 to 45 cycles at 92°C for 15 s and 60°C for 1 min. Amplification products were measured and analysed with the ABI Prism 7900 HT sequence detection system and the SDS software (version 1.2; Applied Biosystems, Foster City, CA, USA). TaqMan probes and primers were provided by the assay-on-demand and assay-by-design services, respectively (Applied Biosystems). *53BP1 *1347_1352delTATCCC was analysed using the MGB Eclipse™ Probe System by Epoch Biosciences (Bothell, WA, USA). Allelic discrimination was carried out as recommended by the manufacturers using the following probes: D353E: assay-on-demand C_2944794_10; G412S: VIC-ACTTCAAAGTGGTGAACC, FAM-AACTTCAAAGTAGTGAACC; K1136Q: VIC-GGAGTACTAATAAGGAAA, FAM-CGGAGTACTAATCAGGAAA; 1347_1352delTATCCC: FAM-CACTTCATCCCAT; TET-CACTTCCTATCCCATC. Primers and probes were designed based on GenBank NM_005657 (NCBI) and are available on request. More than 5% of the genotyping results were confirmed by sequencing, and genotype distributions were consistent with Hardy–Weinberg equilibrium.

### Statistical methods

Calculations of Hardy–Weinberg equilibrium, genotype-specific OR, and 95% CI were carried out using a tool offered by the Institute of Human Genetics, Technical University Munich, Munich, Germany [12]. Age-adjusted ORs and corresponding 95% CIs were computed by means of unconditional logistic regression using SAS (Version 8.2; SAS Institute Inc, Cary, NC, USA). Haplotypes were assigned to subjects using the SNPHAP software (see [13]), which also reports the posterior probability of the most likely assignment [14,15].

## Results and discussion

Inactivation of ATM and ATM substrates such as CHEK2 have been shown to predispose to cancer in humans [7]. Along with ATM and CHEK2, 53BP1 is involved in DNA damage response and tumour suppression. Recent studies have shown that 53BP1 and ATM interact in irradiated cells, suggesting that ATM activation is the consequence of the recruitment of ATM to sites of DNA double-strand breaks by 53BP1 [7,9]. Thus, polymorphic variants in *53BP1 *are excellent candidates for cancer susceptibility. We investigated the impact of three nonsynonymous amino acid exchanges in 53BP1 on breast cancer risk. 53BP1 G413S and K1136Q represented promising candidate SNPs, resulting in the replacement of a nonpolar by a polar amino acid. Genotype frequencies of the three 53BP1 polymorphisms between breast cancer cases and control samples were similar, showing no significant association with breast cancer risk (D353E: age-adusted OR = 1.07, 95% CI = 0.81 to 1.43, *P *= 0.62; G412S: age-adjusted OR = 1.22, 95% CI = 0.86 to 1.74, *P *= 0.26; K1136Q: age-adjusted OR = 1.10, 95% CI = 0.82 to 1.47, *P *= 0.53; Table 1). Aditionally, we detected a novel *53BP1 *6 bp deletion, 1347_1352delTATCCC, leading to the loss of an isoleucine and a proline residue at positions 450 and 451, which has not been described previously. Comparing the occurrence of this rare, 6 bp deletion between cases and controls revealed an inverse association with breast cancer risk (OR = 0.61, 95% CI = 0.22 to 1.68, *P *= 0.34; Table 1), but lacking statistical significance.

The haplotype distribution and corresponding posterior probabilities are shown in Table 2. Since every mean posterior probability was higher than 0.9, only the most likely haplotypes were used to evaluate the association with breast cancer risk. Haplotype analysis showed a nonsignificant inverse association of the haplotype 1059C-1234G-1347_1352^-^-3406A with breast cancer risk (age-adjusted OR = 0.63, 95% CI = 0.23 to 1.75, *P *= 0.38; Table 2). The distribution of the remaining haplotypes between breast cancer patients and controls was similar, indicating no significant effect with regard to breast cancer risk. Given our sample size, we had a 90% power to detect an odds ratio of 1.65 (D353E), 1.76 (G412S), and 1.66 (K1136Q), respectively [16]. Contrary to standard case-control association studies, this study comprised predominantly cases selected for family history of breast cancer. The use of unselected cases would have required at least twice the sample size to achieve the same power as in the present study [17,18]. The numbers of cases within the risk groups A1 to E were too low to be studied separately, as the power in these subgroups would have been limited. In addition to the results of this study, one cannot exclude the possibility that common 53BP1 SNPs may affect breast cancer risk. Regulatory polymorphisms, for example polymorphisms that reside in promotor or noncoding regions, have been shown to modify gene transcription, mRNA stability, and processing efficiency, as well as DNA methylation [19,20].

## Conclusion

The three known 53BP1 SNPs – D353E, G412S, and K1136Q – lacked association with breast cancer risk. However, we detected a novel, very rare 6bp deletion, 1347_1352delTATCCC, that showed a statistically nonsignificant inverse association with breast cancer risk. Concerning the latter, a much larger study cohort is required to verify any putative significant effect. Additionally, it would be valuable to investigate a possible functional effect of this *53BP1 *deletion and its impact on other cancers.

## Abbreviations

53BP1 = TP53 binding protein; ATM = ataxia telangiectasia mutated protein; bp = base pairs; CHEK2 = cell cycle checkpoint kinase 2; CI = confidence interval; OR = odds ratio; PCR = polymerase chain reaction; SNP = single nucleotide polymorphism.

## Competing interests

The author(s) declare that they have no competing interests.

## Authors' contributions

All authors listed contributed to the production of this manuscript: RK, PB, BW, and RKS provided genomic DNAs of cases studied and helped to draft the manuscript. BB and KH participated in the design and coordination of the study and critically revised the manuscript. BF and JLB performed statistical analyses. BF carried out the SNP genotyping and wrote the manuscript. All authors read and approved the final manuscript.

## Figures and Tables

**Table 1:** Genotype frequencies of 53BP1 polymorphisms in breast cancer patients and controls

Polymorphism	No. (%) of cases	No. (%) of controls	AOR (95% CI)	*P*
D353E (1059C>G)				
CC	165 (48.1)	453 (47.6)		
GC	148 (43.1)	405 (42.5)		
GG	30 (8.7)	94 (9.9)		
Σ	343	952		
CC vs GC+GG			1.07 (0.81–1.43)	0.62
				
G412S (1234G>A)				
GG	269 (78.7)	760 (80.1)		
AG	67 (19.6)	174 (18.3)		
AA	6 (1.8)	15 (1.6)		
Σ	342	949		
AA+AG vs GG			1.22 (0.86–1.74)	0.26
				
1347_1352delTATCCC				
wt/wt	334 (98.2)	931 (97.4)		
delTATCCC/wt	6 (1.8)	25 (2.6)		
delTATCCC/delTATCCC	0 (0.0)	0 (0.0)		
Σ	340	956		
delTATCCC/wt vs wt/wt			0.61 (0.22–1.68)	0.34
				
K1136Q (3406A>C)				
AA	158 (47.4)	448 (47.8)		
CA	144 (43.2)	396 (42.2)		
CC	31 (9.3)	94 (10.0)		
Σ	333	938		
CC+CA vs AA			1.10 (0.82–1.47)	0.53

AOR, age-adjusted odds ratio; CI, confidence interval; wt, wild type.

**Table 2:** Haplotype distribution of 53BP1 polymorphisms^a ^in breast cancer patients and control individuals

	Cases		Controls		OR^b^	95 % CI	*P*
	
Haplotype 1059C>G-1234G>A-1347_1352^-^/^+^-3406A>C	No. (%)	Mean posterior probability	No. (%)	Mean posterior probability			
CG+A	444 (68.1)	1	1242 (67.5)	1	1	-	-
CG+C	2 (0.3)	1	1 (0.1)	1	n.a.	n.a.	n.a.
CG–A	6 (0.9)	0.90	25 (1.4)	0.99	0.63	0.23–1.75	0.38
GG+A	1 (0.2)	1	1 (0.1)	1	n.a.	n.a.	n.a.
GG+C	125 (19.2)	1	376 (20.4)	1	1.02	0.79–1.33	0.86
GA+C	74 (11.3)	1	195 (10.6)	1	1.15	0.83–1.61	0.41

^a^Polymorphisms D353E (1059C>G), G412S (1234G>A), 1347_1352delTATCCC, and K1136Q (3406A>C). ^b^1059C-1234G-1347_1352^+^-3406A as reference. CI, confidence interval; n.a., not available; OR, odds ratio.
